# Polymorphism and Phase-Transition Thermodynamic Properties of Phenazone (Antipyrine)

**DOI:** 10.3390/molecules30132814

**Published:** 2025-06-30

**Authors:** Dmitrii N. Bolmatenkov, Ilyas I. Nizamov, Andrey A. Sokolov, Airat A. Notfullin, Boris N. Solomonov, Mikhail I. Yagofarov

**Affiliations:** Department of Physical Chemistry, Kazan Federal University, Kremlevskaya Str. 18, 420008 Kazan, Russia; ilyainizamov@kpfu.ru (I.I.N.); andasokolov@kpfu.ru (A.A.S.); ayanotfullin@kpfu.ru (A.A.N.); miiyagofarov@kpfu.ru (M.I.Y.)

**Keywords:** differential scanning calorimetry, fast scanning calorimetry, solution calorimetry, vapor pressure, heat capacity, fusion enthalpy, polymorphism, phenazone, antipyrine

## Abstract

In this work, detailed information on the phase-transition thermodynamics of the analgesic and antipyretic drug phenazone, also known as antipyrine, is reported. It was found that the compound forms two polymorphs. Fusion thermodynamics of both forms was studied between 298.15 K and *T*_m_ using the combination of differential scanning calorimetry and solution calorimetry. The vapor pressures above crystalline and liquid phenazone were measured for the first time using thermogravimetry—fast scanning calorimetry technique. These studies were complemented by computation of the ideal gas entropy and heat capacity and by measurements of the condensed phase heat capacities. On the basis of experiments performed, we derived sublimation and vaporization enthalpies and vapor pressure above liquid and both crystalline modifications of phenazone in a wide range of temperatures.

## 1. Introduction

Phase-transition thermodynamic properties of active pharmaceutical ingredients are important characteristics needed for the optimization of the separation and purification processes [1], prediction and modeling of solubilities in pure solvents [2,3,4,5] and mixtures [6], estimation of stability of crystals and co-crystals [7], and crystallization kinetics studies [8]. Due to the low volatility and limited thermal stability, these characteristics are often unavailable from the direct measurements and are to be estimated using different models [9] and additive schemes [5]. In turn, development and testing of such approaches require reliable experimental data. However, the consistency and reproducibility of experimental phase transition enthalpies of active pharmaceutical ingredients are much lower in comparison with other organic substances [10,11,12,13]. This situation is mostly caused by the absence of proper control of the purity, crystallinity, and polymorphic state of the sample. Thus, the accumulation of the reliable phase-transition characteristics for drug molecules is of both practical and fundamental importance.

Another crucial part of drug characterization is polymorphism. Polymorphic state defines a number of practical properties, mainly melting temperature [14], long-time stability [15], solubility [16], and dissolution rate [17]; in some cases, the difference in the solubilities and dissolution rates for different polymorphs may be in order of magnitude [18]. Furthermore, different polymorphic modifications of the same compound are considered as different active pharmaceutical substances and may be patented independently in some countries [19]. As a result, numerous experimental or theoretical methods for polymorph screening were proposed in recent years [20,21,22]. A number of syntenic approaches to access the new polymorphic crystals were developed or modified [23].

**Figure 1:**
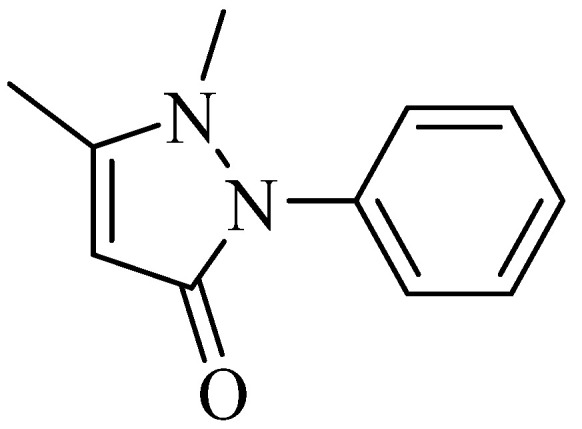
Chemical structure of phenazone (antipyrine).

Phenazone, also known as antipyrine (Figure 1), is one of the drugs in the pyrazolone family [24] used as an analgesic and antipyretic. Being developed at the end of the 19th century, it is still used in medicine, despite the numerous analogs that have been synthesized so far. Some studies are devoted to its solubility [2,5,6,25], crystal [26,27,28] and gas-phase [29] structure, fusion [2,25,30,31,32,33,34,35,36], thermal stability [31], and crystal state heat capacity [37]. Although the data reported in different studies are mostly in good agreement, some inconsistencies between the XRPD (X-ray powder diffraction) pattern in Ref. [38] and in Refs. [26,27,28] is observed. In addition, some melting characteristics reported in Refs. [33,34,35,36] are out of the overall trend from other studies. Moreover, no data on phenazone vapor pressures (except the single-temperature value P (592 K) = 23.2 kPa [39]), sublimation/vaporization enthalpies, and liquid and ideal gas heat capacities, as well as mention of polymorphism, can be found.

The aim of this study is the complete characterization of phase-transition thermodynamics of phenazone in crystalline, liquid, and gaseous states. For the first time, we unambiguously established the existence of two phenazone polymorphs with melting points of 384 K and 376 K. Their enthalpies of fusion, vapor pressures above the crystal and liquid, vaporization/sublimation enthalpies, and crystal, liquid, and ideal gas state heat capacities were obtained. On the basis of the performed studies, temperature dependences of the vapor pressures and fusion, vaporization and sublimation enthalpies were found between 298 K and 425 K. The consistency between the results of different methods demonstrates the relevance of the obtained data.

## 2. Results

Experimental values of the fusion enthalpies (Table S1), crystal and liquid heat capacities (Table S2), vapor pressures (Table S3), and solution enthalpies (Table S5) are provided in the Supplementary Material. Computed ideal gas properties are provided in Tables S11–S13. In the subsections below, we showed experimental evidence of phenazone polymorphism (Section 2.1), reported condensed phase heat capacities (Section 2.2), ideal gas properties (Section 2.3) and saturated vapor pressures (Section 2.4).

### 2.1. Polymorphism and Thermal Behavior

In this work, we revealed the existence of two polymorphic modifications of Phenazone, I and II. Our conclusions are based on XRPD and DSC data.

**Figure 2:**
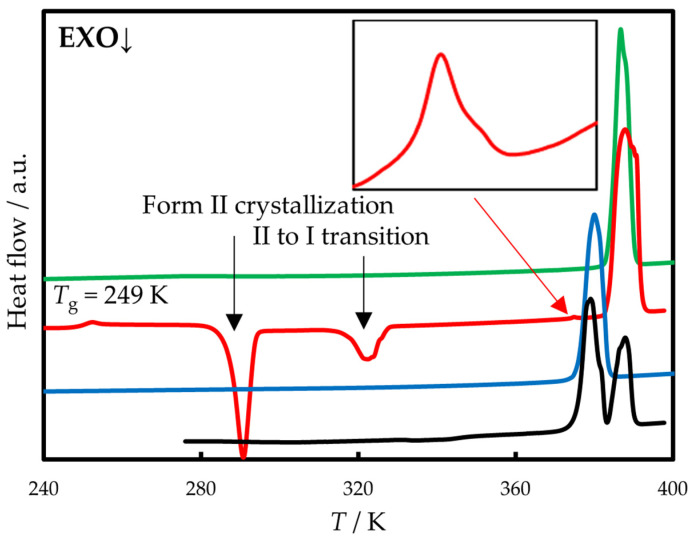
DSC curves of phenazone sample recorded at 10 K min^−1^ after different thermal treatments. Green line—commercial sample (form I); red line—sample cooled to 233 K with the rate of 200 K min^−1^ (glass crystallizes into form II and then almost completely converts into form I); blue line—sample cooled to 293 K with the rate of 200 K min^−1^ and isothermally crystallized in 1 day (form II); black line—sample cooled to 293 K with the rate of 200 K min^−1^ and isothermally crystallized in 5 min (mixture of forms I and II).

DSC thermograms of phenazone are shown in Figure 2. A commercial sample existing in form I melts at 384.1 K. Its fast cooling (200 K min^−1^) to 243 K leads to glassy compound. Subsequent heating at 10 K min^−1^ reveals glass transition (*T*_g_ = 249 K, lit. 256 K [40] and 251 K [32]) and two exothermic events at 280–295 K and 320–330 K. Further, two melting peaks can be observed: the small peak at 376.2 K (form II, see inset in Figure 2) and the main peak at 384.1 K (form I).

Polymorphs I and II can be crystallized from the melt separately. Cooling of the melt to 310 K and its subsequent heating at 10 K min^−1^ leads to cold crystallization around 330 K and melting of form I identical to the commercial sample. Slightly above 330 K, phenazone has a low crystallization tendency, that enabled us to measure the liquid heat capacity in the range of existence of supercooled liquid (355–385 K). Pure form II can be obtained by fast cooling (200 K min^−1^) of the melt to 293 K and subsequent isothermal crystallization over 1 day. Smaller annealing times lead to the formation of a mixture of polymorphs I and II (Figure 2, black line). After each DSC scan, the sample phase composition was monitored using XRPD.

Our observation shows that the exothermic event found in the temperature range 280–295 K is associated with the crystallization of form II. In its turn, the exothermic peak at 320–330 K may be associated with both crystallization of form I and II to I transition. Form II is a metastable one (see Section 3.1) and has a tendency to transform to form I in the presence of form I. The extent of transformation varied between the experiments and was hard to control. The enthalpy of the exothermic event at 320–330 K (Figure 2, red line) equals −4 kJ mol^−1^; the same difference in the enthalpies of forms II and I at this temperature can be calculated using Equation (4). However, a peak at 320–330 K is also observed if the sample was cooled to 310 K and heated up. In this case, the crystallization of melt leads to the formation of pure form I.

**Figure 3:**
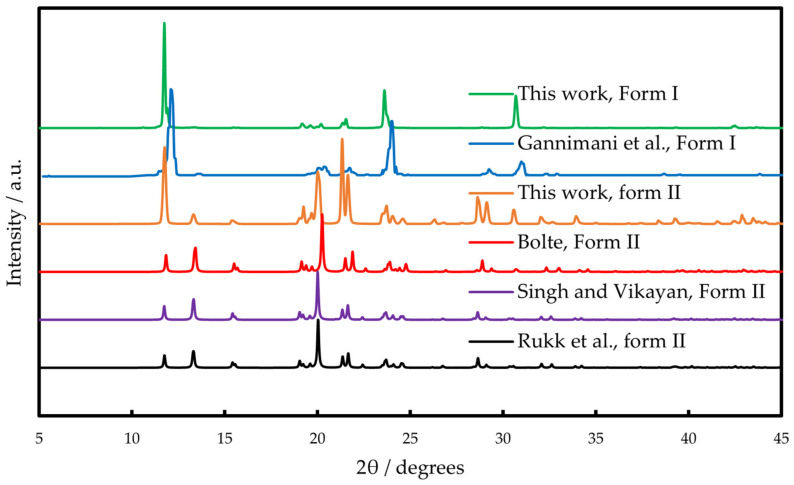
XRPD spectra of phenazone polymorphs I and II. The data from Gannimani et al. [38] were extracted from the graph using build-in digitizer function in the Origin 2018 Pro. The data from Bolte [28], Singh and Vijayan [27] and Rukk et al. [41] were calculated based on single-crystal data using Mercury 4.2.0 Software.

For both polymorphs, XRPD spectra were recorded and compared with the available literature data. Single-crystal structure analysis of phenazone was reported by Romain [26], Singh and Vijayan [27], Bolte [28] and Rukk et al. [41]. All of these studies demonstrate that phenazone crystallizes in C2/c phase group; unit cell parameters determined in these works agree well, as can be seen from Table 1. XRPD spectra obtained in this work for the form II crystallized from the melt at 293 K are identical to those calculated from single-crystal data from Refs. [27,28,41] (Figure 3). In Ref. [38], authors reported XRPD spectra of phenazone. This pattern is similar to that obtained in this work for the commercial sample and corresponds to form I (Figure 3). The melting point of Form I of 384 K was reported in Ref. [38].

In Ref. [27], crystal was obtained by slow evaporation of aqueous solution at room temperature. This procedure may be more convenient for large-scale production of form II in comparison with melt crystallization. No details of crystal preparation can be found in Refs. [26,28,41].

### 2.2. Condensed Phase Heat Capacities

**Figure 4:**
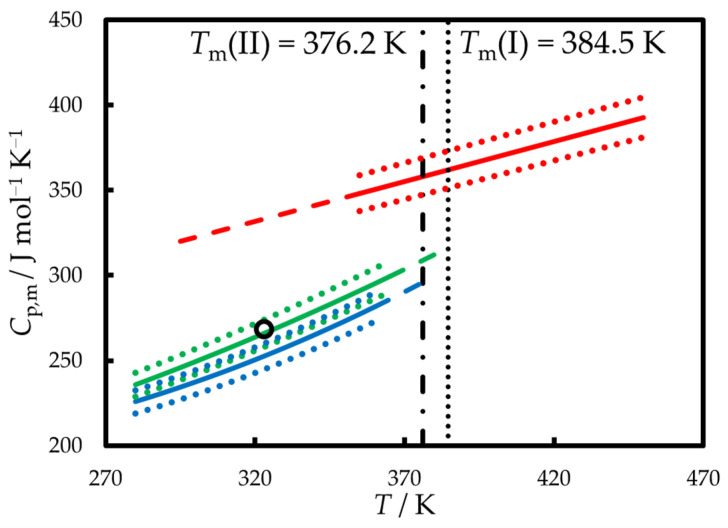
The heat capacities of phenazone measured in this work and available from the literature. Green line—form I, this work; blue line—form II, this work; red line—liquid, this work; black point—form I, Satoh and Sogabe [37]. Dotted lines are uncertainty limits. Dashed lines represent the results of extrapolation. Vertical lines represent the melting points of form I and II.

The heat capacities of I and II polymorphs and liquid phenazone are shown in Figure 4. *C*_p,m_(cr) was also measured by Satoh and Sogabe [37]; however, polymorphic state was not specified. *C*_p,m_(cr, 323 K) = 268.2 J mol^−1^ K^−1^ determined in [37] agrees well with that of form I at the same temperature (266.1 J mol^−1^ K^−1^), as determined in this work.

*C*_p,m_(*T*) measured in this work were fitted by the following functions of temperature:

*C*_p,m_(cr, form I, *T*) = 139.2 + 3.676·10^−2^·(*T/*K) + 1.102·10^−3^·(*T/*K)^2^ *U*_r,tot_ < 0.03

*C*_p,m_(cr, form II, *T*) = 236.1 − 0.6069·(*T/*K) + 2.036·10^−3^·(*T/*K)^2^ *U*_r,tot_ < 0.03

*C*_p,m_(l, *T*) = 182.2 + 0.4678·(*T/*K) *U*_r,tot_ < 0.03

*U*_r,tot_ is total relative expanded uncertainty (0.95 level of confidence, *k* ≈ 2), including the reproducibility of the measurement, calibration, and fitting uncertainty.

In Ref. [34], $\Delta_{\mathrm{cr}\;I}^{l}C_{p,m}(390\;K)$ = 24.5 J mol^−1^ K^−1^ was reported (the polymorphic state was assumed to be I on the basis of melting properties). This value is comparable with $\Delta_{\mathrm{cr}\;I}^{l}C_{p,m}(390\;K)$ = 43 J mol^−1^ K^−1^ found from our measurements, taking into account the combined error of *C*_p,m_ measurements for the two phases.

### 2.3. Optimized Geometry, Ideal Gas Heat Capacities and Entropies

**Figure 5:**
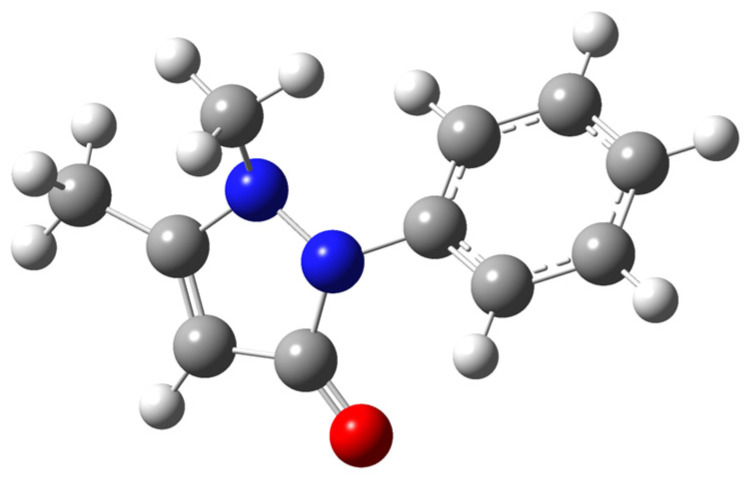
Optimized geometry of phenazone in the gas phase obtained at B3LYP/def2-TZVPP theory level (white—hydrogen, gray—carbon, blue—nitrogen, red—oxygen).

The optimized geometry of phenazone is shown in Figure 5.

The structure and conformational state of phenazone were previously studied by Écija et al. [29] using the MP2 method with a 6-311++G(d,p) basis set. Despite the difference in the theory level, the data obtained in this work are in good agreement with the result presented in Ref. [29]. The only discrepancy is observed in the shape of the potential energy surface of the phenyl group. This is due to the different behavior of the –N–CH_3_ group observed upon rotation of the phenyl group.

Écija et al. reported that, when the phenyl group comes closer to the methyl group, they form a transition state, in which these two groups are arranged in a planar configuration. Then, during the subsequent rotation of the phenyl ring, the methyl group gradually returns to its original position. This results in an energy surface with a peak that slowly decreases towards the energy of the optimized geometry.

In this work, we observed a rapid reorientation of the –N–CH_3_ group as the rotating phenyl group approaches it. It switches to the other side relative to the surface of the five-membered ring, allowing the phenyl group to continue to rotate without steric hindrance. This process repeats when the other part of the phenyl ring comes closer to the methyl group, resulting in the reorientation of the latter back to its original position. Such behavior gives a potential energy scan with two sharp peaks. Due to the symmetry of the phenyl ring, the second half of the PES is identical.

It is interesting to note that the shape of the second part of the potential energy surface presented by Écija et al. coincides with the results of this work and seemingly contains a rapid reorientation of the methyl group.

**Figure 6:**
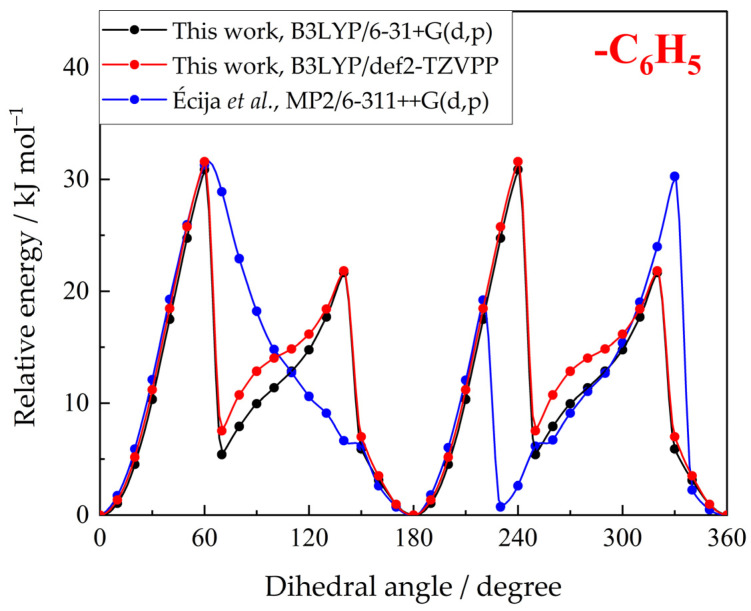
Potential energy surfaces for phenyl group rotation in phenazone obtained in this work at B3LYP/6-31+G(d,p) (black) and B3LYP/def2-TZVPP (red) theory levels and in Ref. [29] (blue).

Despite the differences in the shape of the PES, the torsional barrier heights of phenyl group rotation found in both works are similar. The contributions of internal rotation to the thermodynamic properties of phenazone in the ideal gas state calculated using two potential energy surfaces also differ by only 0.4 J K^−1^ mol^−1^ for heat capacities and 0.2 J K^−1^ mol^−1^ for entropies at maximum. The structural and energetic properties of phenazone obtained in this work are compared with the values provided by Écija et al. [29], as shown in Table 2 and Figure 6.

Comparing the results obtained in this work using two different basis sets revealed insignificant differences. The calculated ideal gas heat capacities and entropies agree within 1.0% and 0.4%, respectively; the obtained potential energy surfaces for internal rotations are also very similar. This shows that the computationally more affordable 6-31+G(d,p) basis set can be used for the determination of the ideal gas heat capacities and entropies with no loss of accuracy. Furthermore, the vibrational contributions to the thermodynamic properties determined at the B3LYP/6-31+G(d,p) theory level could be even more precise due to the application of two scaling factors for vibrational frequencies.

The summary of the ideal gas heat capacities and entropies of phenazone is provided in Tables S11 and S12.

### 2.4. Thermodynamics of Vaporization/Sublimation

**Figure 7:**
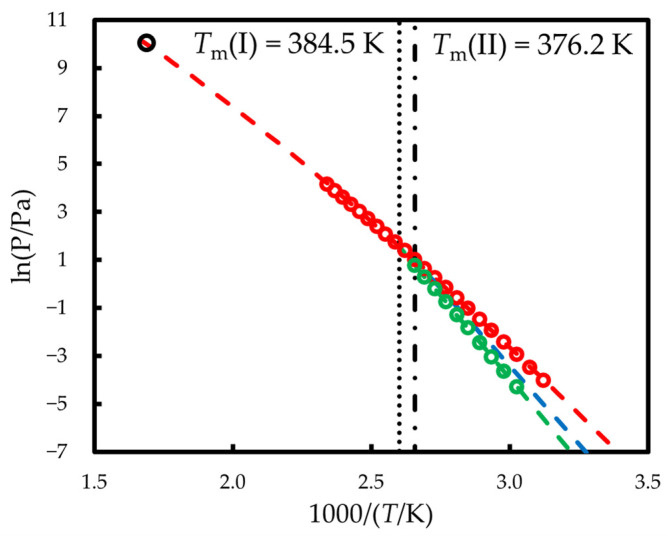
The vapor pressures above crystalline I (green) and liquid (red) phenazone measured in this work using thermogravimetry—fast scanning calorimetry method. Dots are experimental points, and the line is the result of the fit according to Equation (5). Black point is vapor pressure above liquid phenazone at 592 K [39]. Blue line represents the vapor pressures above crystalline form II calculated in this work (see Section 3.2).

The temperature dependences of the saturated vapor pressures above crystalline form I and liquid phenazone are shown in Figure 7. The parameters of Clarke–Glew equation used to fit the data are provided in Table S4. These *p*-*T* curves intersect at *T* = 385.5 K which agrees well with the melting point of form I, 384.5 K. Sublimation (form I) and vaporization enthalpies derived from *p*-*T* dependences assuming ideal gas behavior at the mean temperature of each experiment are equal $\Delta_{\mathrm{cr}\;I}^{g}H(353\;\;K)$ = 114.3 ± 2.6 kJ mol^−1^ and $\Delta_{l}^{g}H(374\;K)$ = 86.4 ± 1.9 kJ mol^−1^ (the enthalpy errors correspond to the standard uncertainties; uncertainty analysis is described in Section S1.6.4 of the Supplementary Material). Being corrected to 384.5 K, they give $\Delta_{\mathrm{cr}\;I}^{g}H(384.5\;\;K)$ = 112.9 ± 2.6 kJ mol^−1^ and $\Delta_{l}^{g}H(384.5\;\;K)$ = 85.4 ± 1.9 kJ mol^−1^. $\Delta_{\mathrm{cr}\;I}^{l}H(384.5\;\;K)$, found as their difference, is 27.5 ± 3.2 kJ mol^−1^, which moderately agrees with the $\Delta_{\mathrm{cr}\;I}^{l}H(384.5\;\;K)$ = 24.0 ± 1.0 kJ mol^−1^ obtained from direct calorimetric measurements.

No data on the vapor pressure above crystalline and liquid phenazone was found in the literature except the single point P (592 K) = 23.2 kPa [39]. The extrapolation of the values measured in this work to 592 K according to Equation (5) leads to P = 21.9 kPa, which agrees with Ref. [39] within 6%.

Previously, Schnitzler et al. using TG-DSC showed that in both nitrogen and oxygen atmospheres the endothermic event accompanied by the mass loss is observed in the range 470–600 K [31]. Authors interpreted these events as decomposition; however, it may be associated with evaporation because the open crucibles were used in the above experiment. Between 470 and 600 K, the vapor pressure of liquid phenazone estimated on the basis of our data changes from 0.5 kPa to 26.0 kPa, which demonstrates its pronounced volatility. A single-temperature vapor pressure measurement reported in [39] and discussed above also indicates that the observed thermal event is vaporization.

The roughly estimated enthalpy of this process is 50 kJ mol^−1^ [31]. A higher value (ca. 70 kJ mol^−1^) of the vaporization enthalpy is expected in the temperature range 470–600 K. However, the data treatment and experimental conditions in Ref. [31] may significantly influence the obtained value that partly explains this disagreement.

## 3. Discussion

This section includes discussion of the fusion (Section 3.1) and sublimation and vaporization (Section 3.2) thermodynamics of phenazone polymorphs between 298.15 K and *T*_m_.

### 3.1. Thermodynamics of Fusion Between 298.15 K and T_m_

The available data on the fusion of phenazone polymorphs are collected in Table 3. Despite the fact that no information on the polymorphic state can be found in Refs. [2,25,30,31,32,34], we assumed that the studied form was form I due to commercial origin and similarity in melting properties. Melting temperatures and enthalpies of fusion available for this form are in good agreement, except $\Delta_{\mathrm{cr}}^{l}H(T_{m})$ = 22.2 kJ mol^−1^ [25] and $\Delta_{\mathrm{cr}}^{l}H(T_{m})$ = 25.18 kJ mol^−1^ [32]. However, these values partly compensate each other. After averaging, $\Delta_{\mathrm{cr}\;I}^{l}H$ = 24.0 ± 1.0 kJ mol^−1^ at 384.5 ± 0.8 K was obtained.

No data on the fusion of form II was found in the literature except the melting points of 378 K [35] and 378.5 K [36], close to the value of 376.2 K measured in this work.

Among others, in Ref. [33], $\Delta_{\mathrm{cr}}^{l}H$ = 28.1 kJ mol^−1^ was reported at an unknown temperature. There is no possibility of distinguishing if there was another polymorph or the reported value had large uncertainty.

The thermochemistry of fusion at 298.15 K was studied by a combination of solution calorimetry and Kirchhoff’s Law of Thermochemistry. On the one hand, $\Delta_{\mathrm{cr}}^{l}H(298.15\;K)$ of forms I and II were found using the respective values $\Delta_{\mathrm{cr}}^{l}H(T_{m})$ and Equation (4). According to this procedure, $\Delta_{\mathrm{cr}}^{l}H(I,\;298.15\;K)$ of 18.6 ± 1.1 kJ mol^−1^ and $\Delta_{\mathrm{cr}}^{l}H(\mathrm{II},\;298.15\;K)$ of 14.6 ± 0.3 kJ mol^−1^ were found. On the other hand, $\Delta_{\mathrm{cr}}^{l}H(I,\;298.15\;K)$ can be estimated using solution calorimetry approach [42]. Within it, the enthalpy of fusion at 298.15 K can be found as a difference between the solution enthalpies of crystalline and liquid compound in the same solvent S (Equation (1)):

(1) $$\Delta_{\mathrm{cr}}^{l}H^{A}(298.15\;K)=\Delta_{\mathrm{soln}}H^{A/S}(\mathrm{cr},\;298.15\;K)-\Delta_{\mathrm{soln}}H^{A/S}(l,\;298.15\;K)$$

where $\Delta_{\mathrm{soln}}H^{A/S}(\mathrm{cr},\;298.15\;K)$ and $\Delta_{\mathrm{soln}}H^{A/S}(l,\;298.15\;K)$ are the solution enthalpies of crystalline and liquid compound A in the solvent S at 298.15 K.

While $\Delta_{\mathrm{soln}}H^{I/\mathrm{DMF}}(298.15\;K)$ was measured in this work (Table S5), $\Delta_{\mathrm{soln}}H^{A/S}(l,\;298.15\;K)$ may be easily estimated [43]. In our previous studies [44,45] we used *N,N*-dimethylformamide (DMF) as a solvent for aromatic amides. Phenazone and DMF do not exhibit specific interactions. The hydrazide entity of the phenazone molecule is structurally similar to DMF. On the other hand, the solution enthalpy of liquid aromatic compounds in DMF does not strongly deviate from 0; particularly, for $\Delta_{\mathrm{soln}}H^{\mathrm{Benzene}/\mathrm{DMF}}(298.15\;K)$ = 0.2 kJ·mol^−1^ [46]. Thus, similar to the previous studies of non-hydrogen-bonded “like dissolves like” systems [43], the solution enthalpy of liquid phenazone in DMF at 298.15 K is expected to be in the range of 1 ± 1 kJ·mol^−1^. Combining $\Delta_{\mathrm{soln}}H^{I/\mathrm{DMF}}(298.15\;K)$ of 20.4 ± 0.3 kJ·mol^−1^ and this estimate, one can obtain $\Delta_{\mathrm{cr}}^{l}H^{I}(298.15\;K)$ = 19.4 ± 1.0 kJ·mol^−1^, agreeing with the above value of 18.6 ± 1.1 kJ mol^−1^.

**Figure 8:**
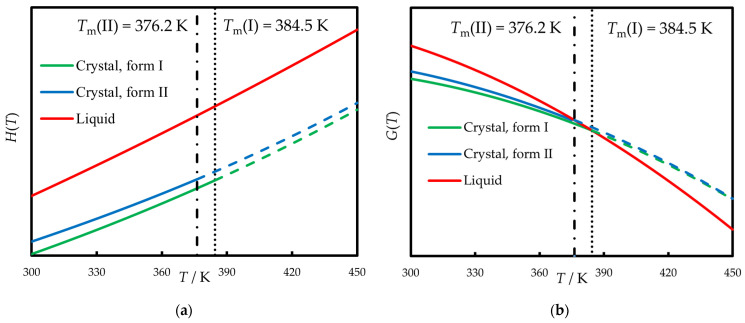
(**a**) Schematic *H*(*T*) diagram for phenazone polymorphs; (**b**) schematic *G*(*T*) diagram for phenazone polymorphs. *G*(*T*) of form I and II intersect at 450 K, *G*(*T*) of liquid and form I intersect at 384.5 K, and *G*(*T*) of liquid and form II intersect at 376.2 K. Dashed lines are result of extrapolation above melting points. Melting points are shown by dotted and dashed vertical lines.

The knowledge of the melting parameters and heat capacities enables the comparison between the relative energies of polymorphs and liquid phenazone. Schematic temperature dependence of enthalpy (*H*(*T*)) and Gibbs energy (*G*(*T*)) for all forms is shown in Figure 8. The right diagram shows that below *T*_m_ form I always has the lower Gibbs energy than form II. The intersection point of these lines is about 450 K, where both forms are unstable compared with liquid phenazone. Thus, polymorph II is the metastable one. However, our studies evidence that form II has no tendency to transform into form I at least within one week under ambient conditions.

### 3.2. Thermodynamics of Vaporization/Sublimation at 298.15 K

The vapor pressures above crystalline form II were not measured because of difficulties in obtaining pure form II on the surface of UFS1 chip sensor. *p*-*T* curve for this form was calculated using its melting characteristics and the data for liquid phenazone because of their lower uncertainty and wider range of measurements. The obtained results are provided in Table 4 and shown in Figure 7 (blue line).

The vapor pressures above crystalline form I and liquid phenazone were extrapolated to 298.15 K according to the Clarke–Glew equation. Table 5 represents obtained values together with the vaporization and sublimation enthalpies at 298.15 K.

## 4. Materials and Methods

### 4.1. Materials

Phenazone (C_11_H_12_N_2_O, CAS No. 60-80-0) was purchased from Weifang Economic Zone Hotspot, Biotechnology Center, China, in form I. The purity was determined using HPLC with pure acetonitrile as an eluent and was found to be 0.9954 (mole fraction). The compound was used without further purification. Form II of phenazone was obtained through melt crystallization in DSC crucibles, as described in Section 2.1. Polymorphic purity of each form was confirmed by DSC and XRPD (Section 2.1). Absence of any decomposition was checked by HPLC analysis after DSC measurements. The data on the phenazone and other compounds used is summarized in Table 6.

### 4.2. Differential Scanning Calorimetry

The heat capacities and enthalpies of fusion and polymorphic transition were determined using power compensation DSC 8500 (Perkin Elmer, Waltham, MA, USA). All experiments were performed in a nitrogen atmosphere (Nurgas, Kazan, Russia; volume fraction of nitrogen >0.99999) with a flow rate of 30 mL min^−1^. Before each experiment, aluminum crucibles were annealed at 423 K for 5 min.

Polymorphic behavior was studied by heating and cooling the samples between 240 K and 400 K at 10 K min^−1^ (Section 2.1). Form II was obtained by fast cooling (200 K min^−1^) of molten phenazone to 293 K and subsequent isothermal crystallization during 1 day.

During the enthalpy measurements, samples with a mass of 5–10 mg were heated from 273 K to 398 K at a rate of 10 K min^−1^. In the heat capacity measurements, samples with a mass of 5–10 mg were studied using a three-step procedure, including measurement of two isothermal and one dynamic segment (10 K min^−1^) for an empty crucible, standard sapphire disk and sample. The data evaluation was performed using Pyris Software (Version 13.4). An accuracy of the heat capacity measurements was verified using crystalline anthracene, thioxanthone, and indium as standards; deviation between measured and reference values did not exceed ±1.7% (Table S13). In the verification procedure performed according to Ref. [47] the literature data from Refs. [48,49,50] were used.

### 4.3. Fast Scanning Calorimetry

The vapor pressures of crystalline I and liquid phenazone were measured using thermogravimetry—fast scanning calorimetry method [51]. Experiments were performed using Flash DSC1 (Mettler Toledo, Greifensee, Switzerland) in nitrogen atmosphere (Nurgas, Kazan, Russia; volume fraction of nitrogen >0.99999) with a flow rate of 40 mL min^−1^ at the sensor support temperature (SST) of 303 K. The UFS1 chip sensor was prepared and calibrated as described previously [52].

Experiments were performed for the samples with a mass in the range of 50–300 ng, which were placed on the surface of USF1 chip sensor using the copper wire. Before the measurements, the sample was heated slightly above its melting temperature to obtain reproducible thermal contact. Phenazone had no tendency to crystallize on the chip sensor, so the compound was studied below its melting temperature in the supercooled liquid state. To obtain crystalline form I, crystallization was induced by adding a small crystal of form I to the molten sample. In the case of form II, such procedure led to the formation of the mixture of polymorphs. Therefore, sublimation was studied for polymorph I only.

**Figure 9:**
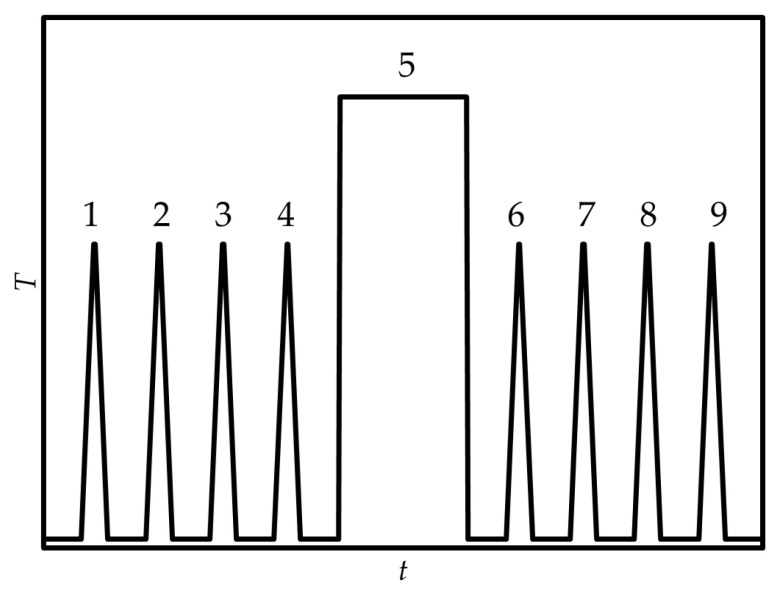
Temperature program used for vapor pressure measurements by thermogravimetry—fast scanning calorimetry method [51].

The temperature program (Figure 9) included registration of the absolute heat capacity of the sample (*C*_p_, J K^−1^) by subsequent heating–cooling cycles with a rate of ±1000 K s^−1^ (steps 1–4 in Figure 9), an isothermal evaporation stage (step 5 in Figure 9) and repeated registration of *C*_p_ (steps 6–9 in Figure 9) to determine the amount of evaporated sample:

(2) $$\Delta n=\frac{\Delta C_{p}}{C_{p,m}}$$

where *C*_p,m_ (J mol^−1^ K^−1^) is molar heat capacity, measured in this work by DSC. Rates of transfer to isothermal stage were of ±10,000 K s^−1^, which completely suppresses evaporation during the heating and cooling stages.

The vapor pressures were then calculated as follows:

(3) $$P_{\mathrm{sat}}=-\frac{\Delta n}{\Delta t}\cdot \frac{RT_{\mathrm{vap}}}{S_{\mathrm{vap}}\beta_{c}}\;\;\;$$

where *S*_vap_ (m^2^) is evaporation area, R is gas constant (8.314 J mol^−1^ K^−1^), *T* (K) is isotherm temperature, Δ*t* (s) is isotherm length, and *β*_c_ (m s^−1^) is mass-transfer coefficient. To determine the sample geometry, photographs of the chip sensor were made before and after each evaporation cycle using an optical microscope BX3M (Olympus, Tokyo, Japan) with the 20× lens in reflection mode. The mass-transfer coefficient of phenazone was estimated as proposed in Ref. [51]. Detailed information on the method used and its verification is also provided in Ref. [51].

Measurements of the vapor pressures at each temperature in the range of 330–376 K for crystalline form I and 320–427 K for liquid phenazone were repeated 10 times. The latter interval included the range of the existence of the supercooled liquid state between 320 and 385 K.

The uncertainty analysis for vapor pressures and derived vaporization/sublimation enthalpies is described in Section S1.6 of the Supplementary Material, where methodology proposed in Ref. [53] was used.

### 4.4. Solution Calorimetry

The solution enthalpies of phenazone were determined at 298.15 K using a TAM III precision solution calorimeter (TA Instruments, New Castle, DE, USA). The crystalline compounds were dissolved by breaking a glass ampule containing 25–50 mg of the sample into a glass cell filled with 90 mL of organic solvent (DMFA). The values obtained experimentally were independent of concentration, indicating the obtaining of infinite dilution conditions. The verification of calorimetric system and other experimental features can be found in our previous publication [44].

### 4.5. Computations

Quantum chemistry and statistical thermodynamics were used to calculate the heat capacities and entropies of phenazone in the ideal gas phase between 200 and 600 K. All calculations were performed using the ORCA 6.0 software [54].

Optimized geometry and vibrational frequencies were computed at the B3LYP/6-31+G(d,p) and B3LYP/def2-TZVPP theory levels with tight convergence criteria and D3BJ dispersion correction [55]. The frequencies corresponding to internal rotation were identified according to Ayala [56] and were excluded from further calculations. The remaining frequencies calculated with the 6-31+G(d,p) basis set were scaled by a factor of 0.9795 below 2000 cm^−1^ and 0.9566 above 2000 cm^−1^ as recommended in Ref. [57]. In the case of the def2-TZVPP basis set, a scaling factor of 0.9657 was used for the vibrational frequencies [58].

A 1-D hindered rotor approximation (1-DHR) was implemented for the treatment of the internal rotation according to the algorithm described in Ref. [59]. For each rotating top of phenazone, an optimized scan over 360° with a step size of 10° was performed to obtain the potential energy surfaces (PES). The reduced moments of inertia of the rotating tops were calculated according to Kilpatrick and Pitzer [60]. The Fourier grid Hamiltonian (FGH) method [61,62,63] was implemented for the determination of 500 energy levels of each internal rotation from potential energy surfaces and reduced moments of inertia using software provided by NIST [64]. These energy levels were used for the calculation of the internal rotation contributions to the ideal gas heat capacities and entropies of phenazone.

For the 6-31+G(d,p) basis set, a combination of two scaling factors for vibrational frequencies and the 1-DHR model allows for the calculation of the ideal gas heat capacity, with an average absolute percentage deviation σ_r_ of 1.5% at 300 K and 1.0% at 600 K [65]. In the case of the def2-TZVPP basis set, the deviation is expected to be higher since the same scaling factor is used for the low- and high-frequency vibrations [56].

The computation procedure, all calculated parameters, the heat capacities and entropies of phenazone in the ideal gas phase obtained in this work are available in the Supplementary Material (Tables S6–S12, Figure S2).

### 4.6. X-Ray Powder Diffraction

XRPD spectra of phenazone (Cu Kα radiation, 40 kV, 15 mA) were recorded using MiniFlex 600 diffractometer (Rigaku, Tokyo, Japan) equipped with D/teX Ultra detector. The data were collected at room temperature ranging 2θ from 3° to 50° with a step of 0.02° without sample rotation. The exposure time for each point was 0.24 s.

### 4.7. Temperature Dependence of the Thermodynamic Functions

Phase transition enthalpies measured in this work were adjusted to another temperature (298.15 K or *T*_m_) according to Kirchhoff’s Law of Thermochemistry:

(4) $$\Delta_{\mathrm{phase}\;1}^{\mathrm{phase}\;2}H(T_{2})=\Delta_{\mathrm{phase}\;1}^{\mathrm{phase}\;2}H(T_{1})+\int_{T_{1}}^{T_{2}}\Delta_{\mathrm{phase}\;1}^{\mathrm{phase}\;2}C_{p,m}(T)dT$$

where $\Delta_{\mathrm{phase}\;1}^{\mathrm{phase}\;2}C_{p,m}(T)$ is the difference between the heat capacities of two phases obtained in this work.

The vapor pressures above crystalline and liquid phenazone were fitted using the Clarke–Glew equation:

(5) $$\ln(P/\mathrm{Pa})=\ln(P(T_{0})/\mathrm{Pa})-\frac{\Delta_{\mathrm{cr}/l}^{g}H(T_{0})}{R}(\frac{1}{T}-\frac{1}{T_{0}})+\frac{\Delta_{\mathrm{cr}/l}^{g}C_{p,m}}{R}(\frac{T_{0}}{T}-1+\ln(\frac{T}{T_{0}}))$$

where *T*_0_ is 298.15 K. Since the Clarke–Glew equation does not account for the temperature dependence of $\Delta_{\mathrm{cr}/l}^{g}C_{p,m}$, the mean value in a temperature range between 298.15 K and average vaporization/sublimation temperature was used. Therefore, $\Delta_{\mathrm{cr}/l}^{g}H(T_{0})$, obtained using Equations (4) and (5), may differ insignificantly. In further experiments, phase transition enthalpies derived according to Kirchhoff’s Law of Thermochemistry were used.

## 5. Conclusions

For the first time, the existence of two phenazone (antipyrine) polymorphs, I and II, was unambiguously established, and their thermal properties and relative stability were investigated. Thermodynamic functions of phase changes between two crystalline modifications, liquid, and gaseous phenazone were derived in a wide range of temperatures using a combination of independent experimental and computational methods. Their mutual agreement, as well as agreement with the results of previous studies, evidence the accuracy and reliability of the obtained data.

## Figures and Tables

**Table 1:** Crystallographic parameters of form II phenazone reported in different studies.

Space Group	a/Å	b/Å	c/Å	β/°	Ref.
C2/c ^a^	16.90	7.43	17.83	117.49	[26]
C2/c ^b^	16.919	7.425	17.796	117.03	[27]
C2/c ^c^	16.7125	7.3864	17.5905	116.576	[28]
C2/c ^b^	16.8929	7.4293	17.7768	116.985	[41]

^a^ Measurement temperature was not specified; ^b^ room temperature; and ^c^ measurement temperature is 173 K.

**Table 2:** The comparison of structural and energetic characteristics of phenazone calculated in this work and in Ref. [29] for the ideal gas state.

Property	This Work, B3LYP/6-31+G(d,p)	This Work, B3LYP/def2-TZVPP	Écija et al. [29], MP2/6-311++G(d,p)
*A*/MHz ^a^	1413.1	1427.8	1416.9
*B*/MHz ^a^	491.6	498.6	495.5
*C*/MHz ^a^	397.6	400.4	402.5
*τ*/degree ^b^	54.9	51.7	53.4
*V*(–C_6_H_5_)/kJ mol^−1 c^	30.8	31.6	30.3
*V*(–C–CH_3_)/kJ mol^−1 c^	6.4	6.6	7.6
*V*(–N–CH_3_)/kJ mol^−1 c^	10.9	9.6	10.9
*I*_r_(–C_6_H_5_)/amu Å^2 d^	67.65	66.92	–
*I*_r_(–C–CH_3_)/amu Å^2 d^	3.14	3.12	3.14
*I*_r_(–N–CH_3_)/amu Å^2 d^	3.19	3.16	3.14

^a^ Rotational constants; ^b^ The dihedral angle between the planes of a five-membered ring and a phenyl group; ^c^ Torsional barries heights; ^d^ Reduced moments of inertia of the rotating tops.

**Table 3:** Summary of the enthalpies and temperatures of fusion of phenazone polymorphs. Outliers are shown in *italic*.

Polymorph	*T*_m_/K	$\Delta_{\mathrm{cr}}^{l}H$(*T*m)/kJ mol^−1^	Reference
I ^a^	385	23.9	[2]
I ^a^	385.8	24.52	[30]
I ^a^	383.7	24.5	[31]
I ^a^	384.0	25.18	[32]
I ^b^	384	-	[38]
I ^a^	385.1	22.2	[25]
I ^a^	*389.77*	22.6	[34]
I ^b^	383.9 ± 0.2 ^c^	24.0 ± 0.3 ^c^	this work
I	384.5 ± 0.8 ^c^	24.0 ± 1.0 ^d^	average
II ^b^	376.2 ± 0.3 ^c^	20.5 ± 0.4 ^c^	this work
not stated	-	28.1	[33]

^a^ Polymorphic form is not stated in the original paper and is assumed to be I on the basis of commercial origin, melting point, and enthalpy of fusion; ^b^ confirmed by XRPD; ^c^ expanded uncertainty *U* (0.95 level of confidence, k ≈ 2) includes the reproducibility of measurement and calibration. The initial experimental values are provided in Table S1; ^d^ standard deviation of the literature data and the value obtained in this work.

**Table 4:** Calculated vapor pressures of crystalline phenazone in form II.

*T*/K	*p*(*T*) ^a^/Pa	*T*/K	*p*(*T*) ^a^/Pa
300	4.2∙10^−4^	350	0.21
310	1.7∙10^−3^	360	0.57
320	6.4∙10^−3^	370	1.5
330	2.2∙10^−2^	376.2	2.7
340	7.0∙10^−2^		

^a^ The upper limit of relative standard uncertainty *u*_r_(*p*) is 0.3*p*, which comes from uncertainty of *p* at experimental temperature and uncertainty of extrapolation.

**Table 5:** Vapor pressures and sublimation/vaporization enthalpies of phenazone at 298.15 K.

State	*p* (298.15 K) ^a^/Pa	$\Delta_{\mathrm{cr}/l}^{g}H(298.15\;K)\;/\;kJ\;mol^{-1}$
Crystal, form I	1.4∙10^−4^	116.3 ± 2.7 ^b^
Crystal, form II	3.2∙10^−4^	108.6 ± 2.3 ^c^
Supercooled liquid	1.4∙10^−3^	94.1 ± 2.1 ^b^

^a^ The upper limit of relative standard uncertainty *u*_r_ (*p*) is 0.3*p*, which comes from uncertainty of *p* at experimental temperature and uncertainty of extrapolation; ^b^ combined uncertainty including standard uncertainty of $\Delta_{\mathrm{cr}/l}^{g}H(T_{\mathrm{av}})$ and uncertainty of the heat capacity correction, which is a propagated uncertainty of the heat capacities of the initial and final phases; ^c^ combined uncertainty including standard uncertainties of $\Delta_{l}^{g}H(T_{\mathrm{av}})$, $\Delta_{\mathrm{cr}\;\mathrm{II}}^{l}H(T_{m})$ and uncertainty of the heat capacity correction, which is a propagated uncertainty of the heat capacities of the initial and final phases.

**Table 6:** The origin and purity of compounds used in this study.

Compound	CAS	Supplier	Purity
phenazone	60-80-0	Weifang Economic Zone Hotspot, Biotechnology Center	>0.98 ^a^ 0.9954 ^b^
acetonitrile	75-05-8	Merck	0.9999 ^a^
*N*, *N*-dimethylformamide ^c^	68-12-2	Sigma-Aldrich	0.999 ^a^
sapphire ^d^	1344-28-1	Perkin Elmer	not provided
anthracene	120-12-7	Aldrich	0.999 ^a^
thioxanthone	492-22-8	TCI	0.999 ^a^
indium	7440-74-6	Perkin Elmer	0.99999 ^a^
nitrogen gas	7727-37-9	Nurgas	0.99999 ^e^

^a^ Mass fraction, stated by the supplier; ^b^ Mole fraction, found using HPLC; ^c^ Additionally purified by distillation; ^d^ Sapphire disk provided by Perkin Elmer (USA) as a reference material for the heat capacity measurements; ^e^ Volume fraction of nitrogen stated by the supplier.

## Data Availability

All data are available on request from the corresponding author.

## Supplementary Materials

The following supporting information can be downloaded at: https://www.mdpi.com/article/10.3390/molecules30132814/s1, Table S1: Enthalpies and temperatures of fusion of phenazone polymorphs measured in this work at 0.1 MPa; Table S2: Isobaric heat capacities of crystalline and liquid phenazone measured in this work at 0.1 MPa; Table S3: Values of saturated vapor pressures of crystalline I, supercooled liquid and liquid phenazone measured in this work using thermogravimetry—fast scanning calorimetry and smoothed using Clarke–Glew equation; Table S4: Parameters of Clarke–Glew equation; Table S5: Experimental solution enthalpies of phenazone in DMF measured in this work at 298.15 K and 0.1 MPa; Table S6: Cartesian coordinates of phenazone optimized with B3LYP/6-31+G(d,p); Table S7: Reduced moments of inertia Ir of the rotating tops of phenazone; Table S8: Computed fundamental vibrational wavenumbers of phenazone used in the calculation of the ideal-gas heat capacities; Table S9: Energy levels of hindered rotors encountered in phenazone; Table S10: Contributions of internal rotation to the heat capacity of phenazone; Table S11: Contributions of vibration and internal rotation to the heat capacities of phenazone, as well as isochoric and isobaric heat capacities calculated in this work; Table S12: Contributions of vibration and internal rotation to the entropies of phenazone, as well as entropies themselves calculated in this work; Table S13: Unsmoothed experimental values of molar heat capacities of crystalline anthracene, thioxanthone, and indium used for validation of the performance of the heat capacity measurements; Figure S1: Deviation plot for the Clarke–Glew equation; Figure S2: Potential energy surfaces for internal rotations in phenazone; Equations (S1)–(S8): details of the computations, details of DSC calibration, details of the uncertainty analysis.

## Abbreviations

The following abbreviations are used in this manuscript:

DSC	Differential scanning calorimetry
TG-DSC	Thermogravimetry—differential scanning calorimetry
XRPD	X-ray powder diffraction
HPLC	High performance liquid chromatography
DMF	*N*,*N*-dimethylformamide
