# Supplementary material for: Polymorphism and Phase-Transition Thermodynamic Properties of Phenazone (Antipyrine)
Source: Molecules. 2025 Jun 30;30(13):2814. doi: 10.3390/molecules30132814 (PMC12250850; doi:10.3390/molecules30132814)
Supplement: Supplementary file 1 [file molecules-30-02814-s001.zip › molecules-3718298-supplementary.pdf]

Supplementary Material

Polymorphism and Phase-Transition Thermodynamic Properties of Phenazone (Antipyrine)

Dmitrii N. Bolmatenkov, Ilyas I. Nizamov, Andrey A. Sokolov, Airat A. Notfullin, Boris N. Solomonov[1], and Mikhail I. Yagofarov

Department of Physical Chemistry, Kazan Federal University, Kremlevskaya str. 18, 420008 Kazan, Russia

1.1 Differential scanning calorimetry

Table S1
Enthalpies and temperatures of fusion of phenazone polymorphs measured in this work at 0.1 MPa [a].

mass / mg	polymorph	T_m / K	$\Delta_{cr}^{l}H(T_m)$ / kJ mol^{-1}
7.96	I [b]	384.00	23.84
	I [c]	383.72	24.01
	II [c]	376.21	20.04
7.32	I [b]	384.15	23.91
	I [c]	383.71	23.95
	II [c]	375.94	20.44
9.70	I [b]	384.04	23.88
	I [c]	383.63	23.97
	II [c]	375.98	20.44
7.36	I [b]	384.09	23.88
	I [c]	383.92	24.25
	II [c]	376.45	20.79
6.47	I [b]	384.10	24.02
	I [c]	383.74	23.93
	II [c]	376.43	20.66
**Average**	**I**	**383.9±0.2** [d]	**24.0±0.3** [d]
**Average**	**II**	**376.2±0.3** [d]	**20.5±0.4** [d]

[a] Standard uncertainty *u(p)* = 5 kPa;
[b] Commercial sample;
[c] Sample crystallized from the melt;
[d] Expanded uncertainty *U* (0.95 level of confidence, k ≈ 2), including the reproducibility of the measurement and calibration.

[1] *Correspondence:* boris.solomonov@kpfu.ru

Table S2
Isobaric heat capacities of crystalline and liquid phenazone measured in this work at 0.1 MPa [a].

T / K	$C_{p,m}$ / J mol^{-1} K^{-1}	$U(C_{p,m})$ [b] / J mol^{-1} K^{-1}	T / K	$C_{p,m}$ / J mol^{-1} K^{-1}	$U(C_{p,m})$ [b] / J mol^{-1} K^{-1}
	**crystal, form I**			**crystal, form II**	
280.0	235.9	7.1	330.0	257.6	7.7
285.0	239.2	7.2	335.0	261.3	7.8
290.0	242.6	7.3	340.0	265.1	8.0
295.0	246.0	7.4	345.0	269.1	8.1
300.0	249.4	7.5	350.0	273.1	8.2
305.0	252.9	7.6	355.0	277.3	8.3
310.0	256.5	7.7	360.0	281.5	8.4
315.0	260.1	7.8		**supercooled liquid**	
320.0	263.8	7.9	355.0	348.2	10.4
325.0	267.6	8.0	360.0	350.6	10.5
330.0	271.4	8.1	365.0	352.9	10.6
335.0	275.2	8.3	370.0	355.3	10.7
340.0	279.1	8.4	375.0	357.6	10.7
345.0	283.1	8.5	380.0	359.9	10.8
350.0	287.1	8.6		**liquid**	
355.0	291.2	8.7	385.0	362.3	10.9
360.0	295.3	8.9	390.0	364.6	10.9
365.0	299.5	9.0	395.0	367.0	11.0
	**crystal, form II**		400.0	369.3	11.1
280.0	225.8	6.8	405.0	371.6	11.1
285.0	228.5	6.9	410.0	374.0	11.2
290.0	231.4	6.9	415.0	376.3	11.3
295.0	234.3	7.0	420.0	378.6	11.4
300.0	237.3	7.1	425.0	381.0	11.4
305.0	240.4	7.2	430.0	383.3	11.5
310.0	243.6	7.3	435.0	385.7	11.6
315.0	247.0	7.4	440.0	388.0	11.6
320.0	250.4	7.5	445.0	390.3	11.7
325.0	253.9	7.6	450.0	392.7	11.8

[a] Standard uncertainty $u(p)$ = 5 kPa;
[b] Expanded uncertainty U (0.95 level of confidence, k ≈ 2) of the heat capacity, including the reproducibility of the measurement and calibration.

1.2 Fast scanning calorimetry

Table S3
Values of saturated vapor pressures of crystalline I, supercooled liquid and liquid phenazone measured in this work using thermogravimetry – fast scanning calorimetry and smoothed using Clarke-Glew equation (Eq. 4). Parameters of Eq. 4 are given in Table S4.

T / K	P / Pa	$u(p)$ [a] / Pa	T / K	P / Pa	$u(p)$ [a] / Pa
	**Crystal, form I**			**Supercooled liquid**	
330.5	0.0136	0.0021	350.8	0.374	0.065

335.6	0.0261	0.0041	355.9	0.564	0.102
340.6	0.0472	0.0077	361.0	0.849	0.158
345.7	0.0864	0.0146	366.1	1.26	0.24
350.8	0.157	0.027	371.2	1.89	0.37
355.9	0.276	0.050	376.3	2.72	0.50
361.0	0.474	0.088	381.4	3.92	0.67
366.1	0.814	0.156		**Liquid**	
371.2	1.30	0.26	386.5	5.37	0.94
376.3	2.14	0.43	391.6	7.62	1.38
	**Supercooled liquid**		396.7	11.0	2.0
320.3	0.0170	0.0024	401.8	15.5	3.0
325.4	0.0308	0.0045	406.9	20.9	4.1
330.5	0.0520	0.0079	412.0	27.2	5.5
335.6	0.0883	0.0140	417.1	36.2	7.5
340.6	0.146	0.024	422.2	50.1	10.6
345.7	0.236	0.040	427.3	64.7	14.0

[a] The estimated uncertainties (standard deviation (u)) of vapor pressure include the uncertainty of sample mass determination, sample area determination, the uncertainty of mass transfer coefficient and uncertainty of temperature. Analysis of the uncertainties was made as described in Ref. [1] (see section 1.6 of the Supplementary Material).

Table S4

Parameters of Clarke-Glew equation (Eq. 4). T_0 = 298.15 K.

Phase	$\ln(P(T_0) / \text{Pa})$	$\Delta^{g}_{cr/l}H(T_0)$ / kJ mol^{-1}	$\Delta^{g}_{cr/l}C_{p,m}$ [a] / J mol^{-1} K^{-1}
Crystal I [b]	-8.85388	116.4	-38
Crystal II [c]	-8.03375	108.6	-24
Liquid [b]	-6.57406	93.8	-97

[a] Found as a difference between ideal gas and condensed phase heat capacities obtained in this work;
[b] Fit of the experimental data;
[c] Calculated using the data for liquid phenazone and melting characteristics of form II.

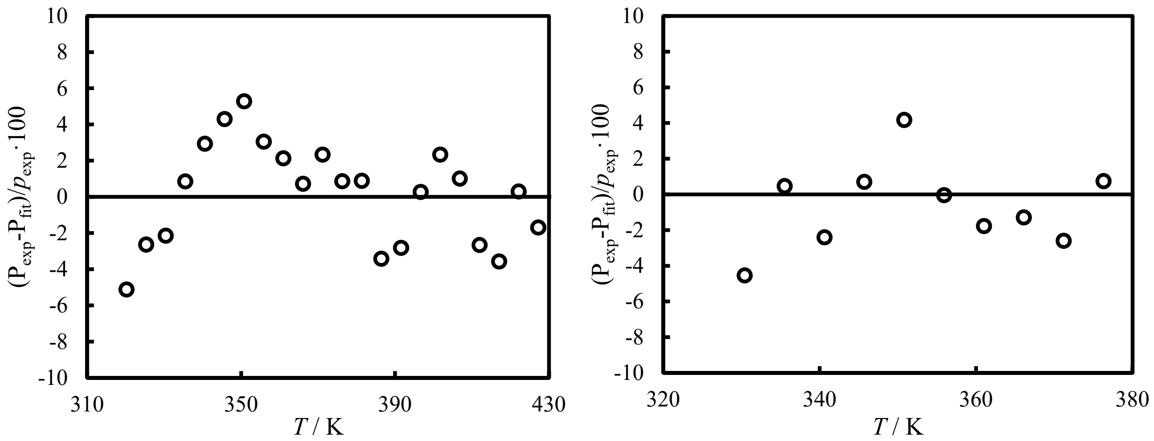

Figure S1. Deviation plot for the Clarke-Glew equation.

1.3 Solution calorimetry

Table S5

Experimental solution enthalpies of phenazone in DMF measured in this work at 298.15 K and 0.1MPa [a].

Mass of sample [b] / mg	Molality [c] / mmol kg^{-1}	$\Delta_{soln}H^{A/S}$ [d] / kJ mol^{-1}
30.97	1.94	20.49
31.72	1.98	21.05
34.68	2.17	19.88
34.92	2.18	20.48
32.31	2.02	20.21
34.07	2.13	20.35
	**Average**	**20.4±0.3**[e]

[a] Standard uncertainties u are $u(T)$ = 0.01 K, $u(p)$ = 5 kPa;

[b] Mass of solute sample which was added in each dissolution experiment;

[c] Molality of solute in solution after experiments. Standard uncertainties u are $u(b)$ = 0.01 mmol·kg^{-1};

[d] Enthalpy of solution of each experiment;

[e] Expanded uncertainty U (0.95 level of confidence, k ≈ 2), including the reproducibility of the measurement and calibration.

1.4 Ideal gas properties

Table S6

Cartesian coordinates of phenazone optimized with B3LYP/6-31+G(d,p) and B3LYP/def2-TZVPP.

B3LYP/6-31+G(d,p)				B3LYP/def2-TZVPP			
**Atom**	**X, Å**	**Y, Å**	**Z, Å**	**Atom**	**X, Å**	**Y, Å**	**Z, Å**
O	0.379786	2.615532	-0.184719	O	0.476795	-2.548763	0.013054
N	0.375864	0.307786	0.239411	N	0.400893	-0.232203	-0.263281
N	1.313616	-0.739256	0.078501	N	1.306612	0.823893	-0.055354
C	2.530605	-0.128496	-0.223724	C	2.537683	0.235979	0.195493
C	-1.005335	0.022832	0.069397	C	-0.977958	0.004439	-0.085268
C	0.980300	1.549391	-0.113375	C	1.040920	-1.472397	0.000036
C	2.369223	1.211715	-0.376553	C	2.418849	-1.107603	0.269256
C	1.269423	-1.747742	1.146350	C	1.218036	1.887085	-1.054704
C	3.780338	-0.944439	-0.325723	C	3.757331	1.078819	0.324102
C	-1.948509	0.886511	0.644673	C	-1.429702	1.089755	0.665745
C	-1.430883	-1.094681	-0.663502	C	-1.894967	-0.870826	-0.668109
C	-3.309201	0.628460	0.476827	C	-2.793137	1.304909	0.816880
C	-2.796259	-1.350924	-0.805807	C	-3.253670	-0.653451	-0.492738
C	-3.741222	-0.491583	-0.239953	C	-3.711160	0.435680	0.241106
H	3.113459	1.929790	-0.687040	H	3.187445	-1.814812	0.524977
H	0.263856	-2.166034	1.201328	H	1.477713	1.520954	-2.052346
H	1.535141	-1.316478	2.120927	H	1.892625	2.689703	-0.770227
H	1.963368	-2.551169	0.894656	H	0.204790	2.277779	-1.070392
H	3.634084	-1.819794	-0.967358	H	4.580690	0.476435	0.700010

H	4.115240	-1.299230	0.656234	H	3.592829	1.910968	1.009721
H	4.580765	-0.335099	-0.750140	H	4.059296	1.495484	-0.639127
H	-1.609365	1.754193	1.196158	H	-0.714262	1.745409	1.139508
H	-0.696379	-1.742622	-1.129325	H	-1.534943	-1.715800	-1.232200
H	-4.035048	1.305808	0.917181	H	-3.135637	2.149891	1.399019
H	-3.118566	-2.219045	-1.373744	H	-3.959978	-1.339040	-0.940980
H	-4.802080	-0.690544	-0.358165	H	-4.772112	0.603143	0.365028

Table S7

Reduced moments of inertia I_r of the rotating tops of phenazone obtained for structures optimized with B3LYP/6-31+G(d,p) and B3LYP/def2-TZVPP.

**Rotating top**	**I_r / amu Å^2**	
	B3LYP/6-31+G(d,p)	B3LYP/def2-TZVPP
$-C_6H_5$	67.645	66.921
$-C-CH_3$	3.136	3.116
$-N-CH_3$	3.190	3.164

Table S8

Computed fundamental vibrational wavenumbers of phenazone used in the calculation of the ideal-gas heat capacities. The frequencies corresponding to the internal rotation were identified according to Ayala [2] and excluded from the table and further calculations.

B3LYP/6-31+G(d,p)		
**ν / cm^{-1}**	**ν / cm^{-1}**	**ν / cm^{-1}**
68.4	846.6	1430.2
102.9	900.6	1452.7
140.2	958.6	1458.9
185.9	976.4	1460.6
248.6	981.5	1473.9
270.3	990.7	1487.6
293.0	992.5	1498.8
323.3	1030.9	1597.7
381.6	1039.0	1611.8
411.7	1045.1	1615.8
449.8	1087.2	1727.7
498.8	1099.6	2893.3
574.1	1133.8	2910.9
593.7	1158.8	2966.3
606.9	1162.1	2992.9
617.6	1174.9	3007.3
635.7	1210.4	3027.1
687.7	1221.5	3043.8
703.2	1304.9	3053.1
726.2	1324.5	3066.3
753.0	1332.0	3075.3

791.9	1372.7	3100.3
829.6	1399.6	3134.5
B3LYP/def2-TZVPP		
**ν / cm-1**	**ν / cm-1**	**ν / cm-1**
66.0	840.2	1409.4
101.7	897.1	1429.4
139.7	952.7	1436.8
180.1	967.3	1443.5
244.8	972.2	1450.5
270.4	979.3	1465.5
287.9	990.1	1481.8
320.6	1019.6	1572.8
379.3	1027.1	1587.4
407.6	1033.8	1592.4
442.2	1076.2	1704.0
495.5	1083.0	2911.2
572.6	1120.1	2931.9
591.8	1143.6	2981.1
606.1	1148.6	3009.8
615.1	1161.6	3024.8
632.7	1195.4	3043.2
687.4	1206.7	3063.3
702.7	1286.9	3072.6
724.1	1305.4	3086.0
749.4	1317.4	3095.3
792.8	1353.3	3122.3
822.5	1377.9	3152.1

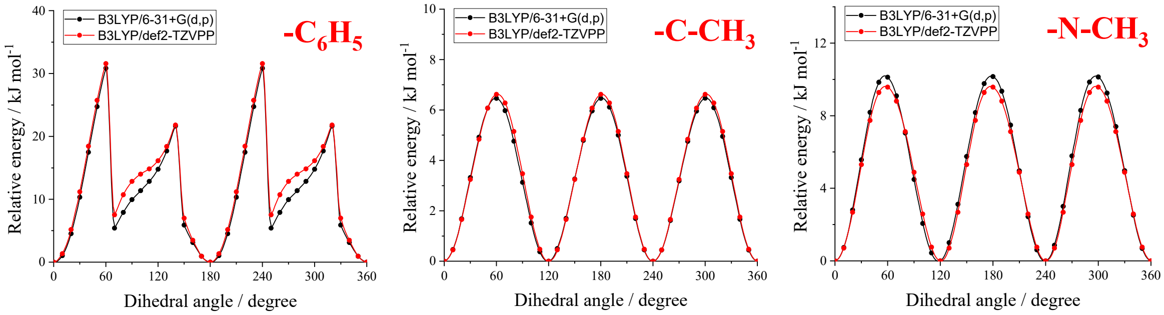

Figure S1. Potential energy surfaces for internal rotations in phenazone obtained at B3LYP/6-31+G(d,p) (black lines) and B3LYP/def2-TZVPP (red lines) theory levels. A dihedral angle of 0 degrees corresponds to the optimal configuration.

Table S9

Energy levels of hindered rotors encountered in phenazone (up to a frequency of 3000 cm-1).

B3LYP/6-31+G(d,p)				
**ν / cm-1**	**ν / cm-1**	**ν / cm-1**	**ν / cm-1**	**ν / cm-1**

		$-C_6H_5$		
25.8	802.3	1323.9	1811.2	2376.4
28.4	831.8	1334.6	1832.0	2408.1
74.3	849.6	1339.9	1848.1	2410.0
76.1	851.3	1346.7	1858.4	2444.1
101.6	859.7	1371.3	1875.9	2444.6
123.8	881.7	1387.3	1886.7	2479.1
157.5	899.8	1393.6	1906.6	2480.3
175.7	917.8	1416.2	1914.6	2513.9
208.1	921.6	1417.4	1940.8	2516.6
225.9	941.0	1437.3	1942.5	2548.8
263.2	957.7	1452.1	1967.8	2552.8
268.8	976.6	1461.7	1971.6	2583.6
299.6	992.3	1484.6	2001.9	2588.9
322.3	995.9	1489.6	2005.6	2618.5
339.1	1011.1	1505.1	2029.0	2624.6
344.5	1035.9	1509.9	2031.2	2653.3
377.0	1045.2	1543.9	2064.6	2659.8
383.8	1060.0	1549.9	2069.3	2688.5
384.6	1063.3	1551.6	2086.6	2694.7
431.4	1091.4	1567.0	2096.3	2724.2
446.9	1095.5	1597.0	2129.8	2729.6
480.0	1108.5	1600.0	2129.9	2760.9
511.6	1134.5	1617.4	2146.1	2765.2
530.4	1136.6	1623.7	2163.0	2798.5
555.4	1155.4	1646.1	2187.4	2801.7
577.6	1157.2	1657.6	2197.2	2837.0
582.4	1182.3	1680.1	2209.4	2839.3
585.2	1205.3	1682.2	2231.4	2876.3
635.7	1205.6	1696.9	2247.0	2877.9
644.7	1215.3	1716.1	2266.2	2916.4
690.2	1228.8	1736.2	2274.9	2917.5
712.8	1251.4	1746.7	2301.2	2957.2
732.4	1275.1	1749.3	2310.3	2957.9
745.7	1276.2	1774.8	2336.6	2998.6
758.5	1276.4	1792.0	2341.9	2999.0
781.7	1295.4	1803.2	2372.2	
		$-C-CH_3$		
77.4	464.6	727.3	1328.1	2009.9
78.4	468.3	823.1	1328.2	2207.4
78.6	480.5	823.2	1482.2	2207.4
224.9	537.1	932.2	1482.3	2415.7
225.9	571.0	932.4	1647.2	2415.8
228.2	573.3	1053.0	1647.3	2634.8
356.1	641.9	1053.0	1823.1	2634.8
360.0	643.2	1185.0	1823.2	2864.7
361.4	724.8	1185.1	2009.9	2864.7
		$-N-CH_3$		

96.8	617.4	927.3	1483.5	2148.2
98.8	620.6	1002.0	1483.6	2341.4
100.4	632.9	1003.6	1633.1	2341.5
286.0	747.6	1104.0	1633.3	2545.5
286.4	757.9	1104.1	1793.9	2545.6
294.6	768.8	1218.4	1794.0	2760.3
460.5	848.9	1218.7	1965.6	2760.3
462.6	854.0	1345.2	1965.6	2985.7
473.3	904.2	1345.3	2148.1	2985.7

B3LYP/def2-TZVPP				
**ν / cm^{-1}**	**ν / cm^{-1}**	**ν / cm^{-1}**	**ν / cm^{-1}**	**ν / cm^{-1}**
		$–C_6H_5$		
25.9	871.7	1420.2	1882.9	2462.0
28.8	885.0	1420.2	1897.7	2475.8
81.9	931.6	1422.3	1916.8	2497.6
82.6	945.5	1440.2	1922.1	2511.6
136.5	974.7	1460.9	1939.6	2533.3
139.5	974.7	1460.9	1952.3	2547.5
192.4	992.0	1483.1	1971.8	2569.1
197.2	1006.7	1501.2	1979.9	2583.5
246.9	1053.0	1505.0	1998.6	2604.8
252.8	1068.2	1505.0	2010.3	2619.6
297.2	1083.2	1543.1	2030.3	2640.3
302.9	1083.2	1551.7	2040.2	2655.7
339.9	1114.4	1551.7	2059.7	2675.2
345.0	1128.7	1561.3	2071.2	2691.9
381.6	1128.7	1600.6	2091.6	2709.6
387.9	1130.2	1600.6	2102.5	2728.3
429.1	1176.0	1602.3	2122.8	2743.3
436.5	1182.5	1620.5	2134.5	2765.3
479.6	1182.5	1651.6	2155.2	2777.0
487.8	1192.3	1651.6	2166.9	2802.8
531.7	1225.7	1660.3	2187.6	2811.3
540.6	1225.7	1678.5	2199.7	2841.0
582.5	1237.8	1704.2	2220.8	2846.9
582.5	1254.6	1704.2	2233.0	2879.9
585.4	1264.4	1717.3	2254.1	2883.7
595.1	1264.4	1735.4	2266.7	2919.4
640.5	1299.5	1757.8	2288.0	2921.9
651.0	1305.2	1757.9	2300.7	2959.5
696.9	1305.2	1773.2	2322.1	2961.1
708.1	1316.7	1791.0	2335.1	
754.3	1345.3	1811.7	2356.7	
766.2	1345.3	1812.2	2369.9	
794.2	1361.1	1828.0	2391.5	
794.2	1378.7	1844.9	2404.9	
812.6	1382.8	1864.4	2426.6	
825.2	1382.8	1866.6	2440.2	

		–C–CH_3		
79.4	474.6	738.4	1342.4	2028.5
80.1	477.0	834.5	1342.5	2227.2
80.1	488.9	834.6	1497.5	2227.3
228.4	548.3	944.3	1497.6	2436.9
232.2	582.0	944.3	1663.5	2436.9
232.3	583.3	1065.7	1663.6	2657.4
363.5	653.1	1065.7	1840.5	2657.4
366.9	653.6	1198.5	1840.6	2888.7
367.8	735.7	1198.5	2028.4	2888.7
		–N–CH_3		
100.0	609.2	901.2	1471.5	2142.9
100.4	614.0	982.8	1471.6	2337.9
100.4	616.0	983.2	1622.8	2338.0
286.0	727.9	1086.6	1622.9	2543.8
291.3	742.8	1086.7	1785.2	2543.9
291.3	745.9	1202.9	1785.3	2760.5
457.9	823.9	1203.3	1958.6	2760.5
463.2	825.6	1331.5	1958.6	2987.9
463.3	885.5	1331.6	2142.8	2987.9

Table S10

Contributions of internal rotation to the heat capacity of phenazone.

***T* / K**	**$C_{v,ir}(T)$ / J K^{-1} mol^{-1}**					
	B3LYP/6-31+G(d,p)			B3LYP/def2-TZVPP		
Rotating top	–C_6H_5	–C–CH_3	–N–CH_3	–C_6H_5	–C–CH_3	–N–CH_3
200	9.0	8.6	8.4	9.4	8.7	8.6
220	9.2	8.5	8.7	9.6	8.6	8.9
240	9.4	8.4	8.9	9.7	8.5	9.0
260	9.5	8.2	9.0	9.8	8.3	9.1
280	9.7	8.0	9.1	10.0	8.1	9.1
298.15	9.9	7.8	9.1	10.2	7.9	9.1
300	9.9	7.7	9.1	10.2	7.8	9.1
320	10.0	7.5	9.0	10.3	7.6	9.0
340	10.1	7.3	8.9	10.5	7.4	8.9
360	10.2	7.1	8.8	10.6	7.2	8.7
380	10.3	6.9	8.7	10.7	7.0	8.6
400	10.3	6.7	8.5	10.8	6.8	8.4
420	10.4	6.6	8.4	10.9	6.7	8.2
440	10.4	6.4	8.2	10.9	6.5	8.1
460	10.4	6.3	8.1	11.0	6.4	7.9
480	10.4	6.2	7.9	11.0	6.2	7.7
500	10.3	6.0	7.8	11.0	6.1	7.6
520	10.3	5.9	7.6	10.9	6.0	7.4
540	10.2	5.8	7.5	10.9	5.9	7.3
560	10.2	5.7	7.3	10.8	5.8	7.1
580	10.1	5.6	7.2	10.8	5.7	7.0

600	10.0	5.6	7.1	10.7	5.6	6.9

Table S11

Contributions of vibration and internal rotation to the heat capacities of phenazone, as well as isochoric and isobaric heat capacities calculated in this work.

B3LYP/6-31+G(d,p)				
**T / K**	**$C_{v,vib}(T)$ / J K^{-1} mol^{-1} [a]**	**$\sum C_{v,ir}(T)$ / J K^{-1} mol^{-1} [b]**	**$C_{v,m}(T)$ / J K^{-1} mol^{-1}**	**$C_{p,m}(T)$ / J K^{-1} mol^{-1}**
200	93.1	26.1	144.1	152.4
220	105.2	26.4	156.6	164.9
240	117.7	26.7	169.3	177.7
260	130.5	26.8	182.2	190.5
280	143.3	26.8	195.0	203.3
298.15	155.0	26.7	206.6	215.0
300	156.2	26.7	207.8	216.1
320	169.0	26.5	220.5	228.8
340	181.7	26.4	233.0	241.3
360	194.1	26.1	245.1	253.5
380	206.2	25.9	257.0	265.3
400	217.9	25.6	268.5	276.8
420	229.4	25.3	279.6	288.0
440	240.4	25.0	290.4	298.7
460	251.0	24.7	300.7	309.0
480	261.2	24.4	310.6	318.9
500	271.0	24.1	320.1	328.4
520	280.5	23.8	329.2	337.5
540	289.5	23.5	338.0	346.3
560	298.2	23.2	346.3	354.7
580	306.5	22.9	354.4	362.7
600	314.5	22.6	362.1	370.4
B3LYP/def2-TZVPP				
**T / K**	**$C_{v,vib}(T)$ / J K^{-1} mol^{-1} [a]**	**$\sum C_{v,ir}(T)$ / J K^{-1} mol^{-1} [b]**	**$C_{v,m}(T)$ / J K^{-1} mol^{-1}**	**$C_{p,m}(T)$ / J K^{-1} mol^{-1}**
200	94.0	26.7	145.6	153.9
220	106.3	27.0	158.2	166.5
240	118.9	27.2	171.0	179.4
260	131.8	27.2	184.0	192.3
280	144.8	27.2	197.0	205.3
298.15	156.7	27.1	208.7	217.0
300	157.9	27.1	209.9	218.2
320	170.8	26.9	222.7	231.0
340	183.6	26.8	235.3	243.6
360	196.1	26.5	247.6	255.9
380	208.3	26.3	259.6	267.9
400	220.2	26.0	271.2	279.5
420	231.6	25.8	282.4	290.7
440	242.7	25.5	293.2	301.5
460	253.4	25.2	303.5	311.8

480	263.6	24.9	313.5	321.8
500	273.4	24.6	323.0	331.3
520	282.8	24.4	332.1	340.4
540	291.8	24.1	340.8	349.2
560	300.5	23.8	349.2	357.5
580	308.8	23.5	357.2	365.5
600	316.7	23.2	364.9	373.2

[a] Vibrational contribution to the heat capacity. Calculated based on the set of frequencies listed in Table S7 using the equation S1.
[b] Sum of the internal rotation contributions for all rotating tops of the molecule.

1.4.1 The procedure of ideal gas phase heat capacities calculation

According to the rigid rotor – harmonic oscillator model, the vibrational contribution to the molar heat capacity in the ideal gas phase can be calculated by Eq. S1 with the set of frequencies from Table S7:

$$C_{\mathrm{v,vib}} = R \cdot \sum_i \frac{(\frac{\Theta_i}{T})^2 \cdot \exp(\frac{\Theta_i}{T})}{(\exp(\frac{\Theta_i}{T})-1)^2}, \tag{S1}$$

where Θ_i is the i-th fundamental vibrational frequency of the molecule.

The contribution of hindered rotation from each rotating top is calculated by Eq. S2 [3] using the energy levels listed in Table S8:

$$C_{\mathrm{v,ir}} = N_{\mathrm{A}} \frac{\left(\sum_j \exp(-\frac{\varepsilon_j}{kT}) \cdot \sum_j \frac{\varepsilon_j^2}{kT^2} \exp(-\frac{\varepsilon_j}{kT})\right) - \left(\sum_j \frac{\varepsilon_j}{kT^2} \exp(-\frac{\varepsilon_j}{kT}) \cdot \sum_j \varepsilon_j \exp(-\frac{\varepsilon_j}{kT})\right)}{\left(\sum_j \exp(-\frac{\varepsilon_j}{kT})\right)^2}, \tag{S2}$$

where ε_j is the frequency of the j-th energy level.

The molar isobaric heat capacity in the ideal gas phase can be found by summing the contributions of vibration ($C_{\mathrm{v,vib}}$), internal rotation of all rotating tops ($\sum C_{\mathrm{v,ir}}$), translation (3/2 R), overall rotation (3/2 R), and the difference between isobaric and isochoric heat capacity (R):

$$C_{\mathrm{p,m}} = C_{\mathrm{v,vib}} + \sum C_{\mathrm{v,ir}} + C_{\mathrm{v,trn}} + C_{\mathrm{v,rot}} + (C_{\mathrm{p,m}} - C_{\mathrm{v,m}}) = C_{\mathrm{v,vib}} + \sum C_{\mathrm{v,ir}} + 4R \tag{S3}$$

Table S12

Contributions of vibration and internal rotation to the entropies of phenazone, as well as entropies themselves calculated in this work.

	B3LYP/6-31+G(d,p)		
***T* / K**	**$S_{\mathrm{vib}}(T)$ / J K^{-1} mol^{-1} [a]**	**$\sum S_{\mathrm{ir}}(T)$ / J K^{-1} mol^{-1} [b]**	**$S(T)$ / J K^{-1} mol^{-1}**
200	67.1	33.9	393.4
220	76.5	36.5	408.5

240	86.2	38.8	423.4
260	96.1	40.9	438.2
280	106.3	42.9	452.8
298.15	115.6	44.6	465.9
300	116.6	44.7	467.2
320	127.1	46.4	481.6
340	137.7	48.1	495.8
360	148.4	49.6	510.0
380	159.3	51.0	524.0
400	170.1	52.3	537.9
420	181.1	53.5	551.7
440	192.0	54.7	565.3
460	202.9	55.8	578.8
480	213.8	56.8	592.2
500	224.7	57.8	605.4
520	235.5	58.8	618.4
540	246.2	59.7	631.3
560	256.9	60.5	644.1
580	267.5	61.3	656.7
600	278.1	62.1	669.1
B3LYP/def2-TZVPP			
**T / K**	**$S_{vib}(T)$ / J K^{-1} mol^{-1} [a]**	**$\sum S_{ir}(T)$ / J K^{-1} mol^{-1} [b]**	**$S(T)$ / J K^{-1} mol^{-1}**
200	66.9	32.9	392.1
220	76.3	35.5	407.2
240	85.9	37.8	422.0
260	95.7	40.0	436.7
280	105.8	42.0	451.3
298.15	115.1	43.7	464.3
300	116.0	43.9	465.7
320	126.4	45.6	480.0
340	137.0	47.3	494.2
360	147.7	48.8	508.3
380	158.4	50.2	522.3
400	169.3	51.6	536.1
420	180.1	52.8	549.9
440	191.0	54.0	563.5
460	201.9	55.1	577.0
480	212.7	56.2	590.3
500	223.6	57.2	603.5
520	234.3	58.2	616.6
540	245.1	59.1	629.5
560	255.7	60.0	642.2
580	266.3	60.8	654.8
600	276.8	61.6	667.2

[a] Vibrational contribution to the entropy. Calculated based on the set of frequencies listed in Table S7 using Eq. (S4).

[b] Sum of the internal rotation contributions for all rotating tops of the molecule.

1.4.2 The procedure of ideal gas phase entropy calculation

According to the rigid rotor − harmonic oscillator model, the vibrational contribution to the entropy in the ideal gas phase can be calculated by Eq. (S4) with the set of frequencies from Table S7:

$$S_{\mathrm{vib}} = R \cdot \sum_i \left(\frac{\frac{\Theta_i}{T}}{\exp(\frac{\Theta_i}{T}) - 1} - \ln\left(1 - \exp(-\tfrac{\Theta_i}{T})\right) \right), \tag{S4}$$

where Θ_i is the *i*-th fundamental vibrational frequency of the molecule.

The contribution of hindered rotation from each rotating top is calculated by Eq. (S5) [3] using the energy levels listed in Table S8:

$$S_{\mathrm{ir}} = R \ln\left(\frac{1}{\sigma_{\mathrm{r}_i}} \Sigma_j \exp(-\frac{\varepsilon_j}{kT}) \right) + \frac{N_{\mathrm{A}}}{T} \frac{\Sigma_j \varepsilon_j \exp(-\frac{\varepsilon_j}{kT})}{\Sigma_j \exp(-\frac{\varepsilon_j}{kT})}, \tag{S5}$$

where σ_{r_i} is the internal symmetry number of the *i*-th rotating top (σ_{r_i} = 2 for phenyl group and σ_{r_i} = 3 for methyl groups) and ε_j is the frequency of the *j*-th energy level.

The translational entropy of an ideal gas was found using Eq. S6 (M = 188.2 g/mol, $P = 10^5$ Pa):

$$S_{\mathrm{trn}} = R \ln\left[\frac{1}{N_{\mathrm{A}}} \cdot \left(\frac{2\pi MkT}{h^2 N_{\mathrm{A}}} \right)^{3/2} \cdot \frac{RT}{P} \right] + \frac{5}{2} R \tag{S6}$$

The rotational entropy of phenazone was calculated using moments of inertia around principal axes ($J_1 = 2.11 \cdot 10^{-44}$ kg·m², $J_2 = 1.71 \cdot 10^{-44}$ kg·m², $J_3 = 5.94 \cdot 10^{-45}$ kg·m²), the symmetry number of phenazone (σ = 1) and Eq. S7:

$$S_{\mathrm{rot}} = R \ln\left[\frac{\sqrt{\pi}}{\sigma} \cdot \sqrt{J_1 J_2 J_3} \cdot \left(\frac{8\pi^2 kT}{h^2} \right)^{3/2} \right] + \frac{3}{2} R \tag{S7}$$

The entropy in the ideal gas phase can be found by summing the contributions of vibration (S_{vib}), internal rotation of all rotating tops ($\sum S_{\mathrm{ir}}$), translation (S_{trn}) and overall rotation (S_{rot}):

$$S = S_{\mathrm{vib}} + \sum S_{\mathrm{ir}} + S_{\mathrm{trn}} + S_{\mathrm{rot}} \tag{S8}$$

1.5 Verification of the heat capacity measurements

DSC was calibrated according to the manufacturer's recommendation using Indium and Zinc samples provided by manufacturer. Each value (onset temperature and area of the peak) was determined three times. The reproducibilities of heat flow and temperature

calibration (0.95 level of confidence, coverage factor 2) were equal to 1 % and 0.1 K, respectively.

The correctness of determination of the heat capacity was checked by measuring the heat capacity of crystalline anthracene, thioxanthone and indium. The heat capacities were measured using three-step procedure. Program including isotherm (3 min), heating with a rate of 10 K min^{-1} and second isotherm (3 min) was repeated for empty crucible, standard sapphire disc (m = 27.96 mg) and sample with a mass of 5-15 mg.

Measured heat capacities were compared with the recommended values for anthracene [4] and indium [5] and with our previous adiabatic calorimetry data for thioxanthone [6].

To check the possible influence the sample mass on the measured values according to Ref. [7], we varied the mass of anthracene samples from 3.52 mg 10.80 mg. No systematic influence was observed.

Relative deviation between measured and reference values varied from -1.5 % to 1.7 %.

Table S13

Unsmoothed experimental values of molar heat capacities of crystalline anthracene, thioxanthone, and indium used for validation of the performance of the heat capacity measurements. Experimental pressure is 0.1 MPa.

T / K	$C_{p,m}$ (exp) / J mol^{-1} K^{-1}	$U(C_{p,m})^{a}$ / J mol^{-1} K^{-1}	$C_{p,m}$ (lit)^{b} / J mol^{-1} K^{-1}	Relative deviation / %
		**anthracene (cr)/ m = 3.52 mg**		
280	196.9	5.9	196.0	0.5
290	205.2	6.2	204.0	0.6
300	210.4	6.3	211.9	-0.7
310	220.1	6.6	219.8	0.1
320	228.4	6.9	227.6	0.3
330	236.6	7.1	235.3	0.6
340	244.0	7.3	243.0	0.4
350	251.8	7.6	250.6	0.5
360	259.0	7.8	258.2	0.3
370	266.3	8.0	265.7	0.2
380	273.8	8.2	273.1	0.2
390	281.6	8.4	280.5	0.4
400	288.8	8.7	287.8	0.4
		**anthracene (cr)/ m = 6.86 mg**		
280	194.2	5.8	196.0	-0.9
290	202.0	6.1	204.0	-1.0
300	209.9	6.3	211.9	-1.0
310	217.8	6.5	219.8	-0.9
320	226.1	6.8	227.6	-0.7
330	233.4	7.0	235.3	-0.8

340	240.3	7.2	243.0	-1.1
350	247.3	7.4	250.6	-1.3
360	254.3	7.6	258.2	-1.5
370	262.4	7.9	265.7	-1.3
380	270.4	8.1	273.1	-1.0
390	278.4	8.4	280.5	-0.8
400	287.0	8.6	287.8	-0.3
**anthracene (cr)/ *m* = 10.80 mg**				
280	194.5	5.9	196.0	-0.7
290	202.2	6.1	204.0	-0.9
300	211.0	6.4	211.9	-0.4
310	218.7	6.6	219.8	-0.5
320	226.9	6.8	227.6	-0.3
330	234.5	7.1	235.3	-0.4
340	242.0	7.3	243.0	-0.4
350	250.1	7.5	250.6	-0.2
360	257.2	7.7	258.2	-0.4
370	265.0	8.0	265.7	-0.2
380	272.5	8.2	273.1	-0.2
390	280.4	8.4	280.5	0.0
400	287.9	8.6	287.8	0.0
**thioxanthone (cr)**				
280	209.3	6.3	209.9	-0.3
290	214.1	6.5	217.0	-1.4
300	221.6	6.7	224.1	-1.1
310	229.4	6.9	231.1	-0.7
320	237.2	7.1	238.1	-0.4
330	242.2	7.3	245.0	-1.1
340	248.8	7.6	251.9	-1.2
350	255.7	7.8	258.7	-1.1
360	263.1	8.0	265.4	-0.9
370	271.1	8.2	272.1	-0.4
380	280.5	8.4	278.7	0.6
390	289.9	8.6	285.3	1.6
400	296.9	8.8	291.9	1.7
**indium (cr)**				
300	27.0	0.8	26.9	0.2
310	27.1	0.8	27.1	-0.1
320	27.4	0.8	27.3	0.3
330	27.5	0.8	27.5	-0.1
340	27.6	0.8	27.7	-0.4
350	27.9	0.8	27.9	0.1
360	28.1	0.8	28.1	0.3
370	28.4	0.8	28.3	0.4
380	28.6	0.9	28.5	0.6
390	28.8	0.9	28.7	0.7
400	29.0	0.9	28.9	0.5

[a] The uncertainties correspond to expanded uncertainty (U) at a level of confidence of 95%, and coverage factor of 2. $u(p)$ = 5 kPa, $u(T)$ in measurements by DSC is 0.1 K where u is standard uncertainty;
[b] Reference data were taken from Ref. [4] for anthracene, from Ref. [6] for thioxanthone, and from Ref. [5] for indium and smoothed by second-order polynomial.

1.6 Uncertainty of the measurements

1.6.1 Uncertainty of the melting properties

Expanded uncertainty U (0.95 level of confidence, k ≈ 2) of the fusion enthalpy and melting temperature includes calibration uncertainty (1 % for enthalpy and 0.1 K for temperature) and reproducibility of the measurements (0.5-1.5 % for enthalpy and 0.2 K for temperature).

1.6.2 Uncertainty of the heat capacities

Expanded uncertainty U (0.95 level of confidence, k ≈ 2) of the heat capacity includes calibration uncertainty (1 %) and reproducibility of the measurements (1-3 %).

1.6.3 Uncertainty of the vapor pressures

Standard deviation of the vapor pressures was estimated according to Ref. [1]. It includes uncertainty of mass loss rate determination (3 %), uncertainty of evaporation area determination (5 %), uncertainty of temperature (1 K), uncertainty of mass-transfer coefficient (10 %) and reproducibility of measurements (5-10 %). The uncertainty of mass loss rate determination includes reproducibility of heat flow (1 %) and uncertainty of the heat capacity (3 %).

1.6.4 Uncertainty of the vaporization enthalpy

The uncertainty of the vaporization enthalpy was calculated considering the standard uncertainties of pressure and temperature. We neglected effect of temperature-independent contributions to $u_r(p)$ since they do not have impact on the slope in ln$p$ vs. $1/T$ coordinates. We followed a methodology described by Cebe *et al.* [8]. 1000 normally distributed ln$p$ vs. $1/T$ pairs were generated per each experimental data point with the standard deviations corresponding to ln(1.07) = 0.07 for ln$p$ and $1/(T\cdot(T+1))$ for $1/T$. From these data 1000 slopes were determined and the standard uncertainty was calculated from the histogram.

References

1. Buzyurov, A.V.; Nagrimanov, R.N.; Zaitsau, D.H.; Mukhametzyanov, T.A.; Solomonov, B.N.; Abdelaziz, A.; Schick, C. Application of the Flash DSC 1 and 2+ for vapor pressure determination above solids and liquids. *Thermochimica Acta* **2021**, *706*, 179067, doi:10.1016/j.tca.2021.179067.

2. Ayala, P.Y.; Schlegel, H.B. Identification and treatment of internal rotation in normal mode vibrational analysis. *The Journal of chemical physics* **1998**, *108*, 2314-2325.
3. Pfaendtner, J.; Yu, X.; Broadbelt, L.J. The 1-D hindered rotor approximation. *Theoretical Chemistry Accounts* **2007**, *118*, 881-898.
4. Goursot, P.; Girdhar, H.L.; Westrum Jr, E.F. Thermodynamics of polynuclear aromatic molecules. III. Heat capacities and enthalpies of fusion of anthracene. *The Journal of Physical Chemistry* **1970**, *74*, 2538-2541.
5. Grønvold, F. Heat capacity of indium from 300 to 1000 K: Enthalpy of fusion. *Journal of Thermal Analysis and Calorimetry* **1978**, *13*, 419-428.
6. Yagofarov, M.I.; Sokolov, A.A.; Gerasimov, A.V.; Solomonov, B.N.; Stepurko, E.N.; Yurkshtovich, Y.N. Thermodynamic Properties of Thioxanthone between 80 and 540 K. *Journal of Chemical & Engineering Data* **2022**, *67*, 3583-3588.
7. Höhne, G.W.H.; Hemminger, W.; Flammersheim, H.-J. *Differential scanning calorimetry*; Springer: 2003; Volume 2.
8. Cebe, P.; Thomas, D.; Merfeld, J.; Partlow, B.P.; Kaplan, D.L.; Alamo, R.G.; Wurm, A.; Zhuravlev, E.; Schick, C. Heat of fusion of polymer crystals by fast scanning calorimetry. *Polymer* **2017**, *126*, 240-247.
